# The Delicate Balance to Adjustment: A Qualitative Approach of Student’s Transition to the First Year at University

**DOI:** 10.5334/pb.409

**Published:** 2018-04-27

**Authors:** Mikaël De Clercq, Nathalie Roland, Magali Brunelle, Benoit Galand, Mariane Frenay

**Affiliations:** 1 Université catholique de Louvain, BE

**Keywords:** academic achievement, higher education, first year at the university, transition, motivation

## Abstract

First year experience in higher education has been extensively investigated in the literature. Yet, two limitations can be identified out of the literature. The majority of the studies focused on single factor analysis, restraining the multifactorial understanding of adjustment’s determinants. Moreover, the temporal unfolding of the first year at the university has mainly been disregarded, limiting the dynamic framing of adjustment process. To overcome these limitations, the current study used a longitudinal qualitative design in order to grasp the dynamic complexity of adjustment process. Semi-structured interviews were conducted in two steps with 17 freshmen from Science department. The aims were to unfold the constructs at play in student’s adjustment process and the dynamic interplay between them over time. The analyses were grounded into Nicholson’s theoretical framework of transition cycle and the material was analyzed through thematic and sequential analysis. Four themes (*readiness, reaching personal drives, fighting an overwhelming program and becoming a self-regulated learner*) and four different events (*starting up, click, exhaustion and deficiencies accumulation*) were identified in the material disclosing the dynamic nature of adjustment process. An overall reflection on the findings is proposed in the conclusion.

## Introduction

The transition from secondary education to university is a particularly challenging experience for freshmen, requiring them to adjust quickly to this new academic context. Credé and Niehorster (2012, p. 134) defined student adjustment to college as *“the ability to effectively adapt to the various challenges encountered in the new college environment”*. They emphasized the myriad social, academic, personal and institutional challenges of this new context that students have to cope with in order to thrive and perform well at university. A substantial proportion of freshmen do not overcome the challenges of this transition phase and fail the first year or drop out (Torenbeek, Jansen, & Hofman, 2010). This situation has been identified as a pervasive educational concern in higher education because it entails financial and psychological cost for the student, his or her family and society (Gale & Parker, 2012).

As a consequence, the student adjustment process has been often investigated in the literature. Up to now, many variables (e.g. the student’s socioeconomic status, academic skills, self-efficacy beliefs and social support) have been found to be related to academic achievement and persistence (Robbins et al., 2004; De Clercq, Roland, Milstein, & Frenay, 2016). These findings support a conception of academic adjustment as a complex multifactorial process. However, two main limitations impede our understanding of this complex process.

First, few studies have employed a multifactorial approach (Allen, Robbins, & Sawyer, 2010). Variables involved in student adjustment have mainly been studied separately. The impact of these variables has been investigated independently of each other, without considering that they are intricately nested in a dynamic process (Busato, Prins, Elshout, & Hamaker, 2000; De Clercq, Galand, & Frenay, 2013). Such a single-factor approach is limited because it does not take into account the interrelationships between the variables. De Clercq and colleagues (2016) emphasized the inadequacy of this approach and assumed that the effect of a variable will depend on the way it interacts with other variables included in the adjustment process. The development of a more global approach could therefore lead to additional clarification of the conditions that foster retention and achievement at university.

Second, the temporal and dynamic nature of academic adjustment is left out of the picture. According to several authors, adjustment has an important temporal nature because the first year at the university is punctuated by several important moments that impact the way the student will adapt to the context (Sauvé, Debeurme, Martel, Wright, & Hanca, 2007). For example, some authors have shown that the first weeks at university are decisive for the student’s trajectory in the program (Astin, 1993; Hurtado, Carter, & Spuler, 1996; Upcraft & Gardner, 1989; Wasylkiw, 2016). Another key moment often discussed in the literature is the announcement of exam results. These results give the student important feedback on his or her ability to succeed and could lead him to question his or her study choice and persistence (Dupont, De Clercq, & Galand, 2015). Scrutiny of the temporal nature of adjustment throughout the first year could therefore provide additional clarification of this thorny issue.

According to Zittoun (2008, 2009), one way to overcome these two limitations would use a qualitative design for the investigation of academic transition, combining real-time and post-hoc data. Real-time data collection examines the student’s spontaneous explanation of his or her experience as it actually unfolds, while post-hoc data offers access to the student’s reconstructed evaluation in the context of a given time-frame (Zittoun, 2009). The qualitative nature of the data fully takes into account the complexity of adjustment (multifactorial approach; Willig, 2013). Moreover, the combination of real-time and reconstructive interviews could make it possible to capture the time dimension of a process by following this process over the course of the academic year (temporal consideration; Zittoun, 2009). The present study therefore employs a qualitative combined design to investigate the multifaceted and temporal adjustment process. In order to start from a specific interpretative viewpoint of student discourse, the analysis was theoretically grounded in Nicholson’s Transition Cycles model (1990) and self-determination theory (Deci & Ryan, 2000).

### From the Nicholson Transition Cycle to Higher Education Literature

**Figure 1:**
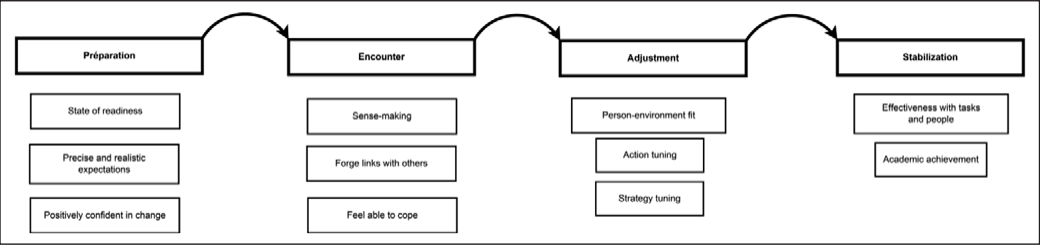
Nicholson’s Transition Cycle (1990) adapted to the context of first year at the university.

Nicholson’s Transition Cycles model (1990) is central to the conception of first-year experience as a dynamic and multifactorial process. This model has attracted a great deal of attention in the literature tackling the issue of transition periods (Crider, Calder, Bunting, & Forwell, 2015). Nicholson claims that the successful achievement of a transition will be determined by four successive stages: preparation, encounter, adjustment and stabilization, each of them characterized by specific tasks and pitfalls (Nicholson & West, 1989). This model initially focused on career changes. However, several authors have broadened its scope so as to address first-year university experience by means of this specific framework (Harris, 2014; Jansen, André, & Suhre, 2013; Polach, 2004; Purnell, 2002). Purnell argued that *“it is the multifaceted nature of transition to university that makes it a fascinating one to study, and Nicholson’s transition cycle appears to provide an excellent framework for this exploration of student experience”* (2002, p. 2). The dynamic nature of the model makes it a sound framework to enhance our understanding of the first-year experience. Moreover, the model is theoretically endorsed by several recent studies that have sought to go beyond the static consideration of academic transition (Coertjens, Brahm, Trautwein, & Lindblom-Ylänne, 2017; Harris, 2014; Jansen et al., 2013; Kyndt, Donche, Trigwell, & Lindblom-Ylänne, 2017, Torenbeek et al., 2010). However, Nicholson’s Transition Cycles model has barely been tested in the higher education context. There is therefore a need for further empirical investigation of this model in order to increase its contextual validity. Following this line of work, the present study has been grounded in this dynamic conception of transition and in empirical literature on freshman academic achievement. In addition, self-determination theory was included as a complementary lens of understanding, which provides a more precise description of the motivational nature of the first-year experience. Figure 1 illustrates the dynamic model of the first-year experience.

#### Preparation Stage

The first stage of Nicholson’s cycle is *preparation*, which takes place before entrance to university. According to Nicholson (1990), the core tasks of preparation are achieving a state of readiness, developing precise and realistic expectations and being positively motivated to change. Completion of these tasks could make the management of the next stages easier (Nicholson, 1990). On the other hand, a number of pitfalls may also be encountered: unreadiness, reluctance and fearfulness.

The tasks and pitfalls described by Nicholson draw on constructs widely studied in the literature on the first year at university. *Readiness* can be defined as preparation in terms of knowledge and skills. It can be related to research on the positive impact of past performance on academic achievement and persistence. (DeBerard, Spielmans, & Julka, 2004; DaDeppo, 2009). As an illustration of the strong impact of this factor, a meta-analysis by Richardson and colleagues (2012) identified a corrected average correlation of .41 between past performance and academic achievement.

Some authors also address the specific question of students’ *expectations* at university (Torenbeek et al., 2010) and the importance of the freshman study choice process in establishing them (Germeijs, Luyckx, Notelaers, Goossens, & Verschueren, 2012; De Clercq et al., 2016). Research has shown that students who make an informed and thoughtful study choice express more satisfaction with respect to the program, engage more deeply in the understanding of the courses and perform better (Biémar, Philippe, & Romainville, 2003; Simons, Dewitte, & Lens, 2004).

Finally, some authors relate Nicholson’s concepts of *positive motivation to change* and fearfulness to the construct of self-efficacy beliefs (Harris, 2014; Jansen & Van der Meer, 2012) Academic self-efficacy beliefs are considered to be one of the most important psychosocial predictors of academic achievement (Elias & MacDonald, 2007). Several studies provided corroborative evidence that confidence in one’s ability and chances of success fosters adaptation to the university through its positive effect on motivational, social, emotional, cognitive and behavioral factors (De Clercq, Dupont, Galand, & Frenay, 2013).

#### Encounter Stage

*The Encounter* stage takes place in a narrow time frame. It occurs during the very first weeks in the new environment and is characterized by the early stages of sense-making (Nicholson & West, 1989). During the encounter phase, freshmen have to adjust their initial beliefs, knowledge and perceptions to the actual academic context. In doing so, three tasks need to be carried out: acquire a sense of one’s ability to cope, undertake the challenge of sense-making, and forge links with others (Harris, 2014; Nicholson, 1990).

A number of connections can be made between Nicholson’s theoretical premises and the three fundamental human needs for autonomy, competence and relatedness of self-determination theory (Deci & Ryan, 2000). The main premise is that the experience of competence, autonomy and relatedness is of paramount importance to the student’s academic engagement, retention and achievement in higher education (Bowman & Denson, 2014; Kahu, 2013; Morrow & Ackermann, 2012).

The need for competence draws on the need to experience oneself as competent within the academic context (Wigfield, Eccles, Schiefele, Roeser, & Davis-Kean, 2006). In this respect it may easily be linked to the task of feeling competent to cope with the context. Research argues that the need for competence is of the utmost importance for a student’s achievement and retention in higher education (Dupont et al., 2015).

The need for autonomy refers to the student’s need for self-determined behavior initiated according to personal desires rather than being controlled by others (Deci & Ryan 2000). This concept encompasses different sorts of internalized and self-determined motivation such as enactment of a learning activity for its own sake or to serve a personally endorsed goal (Vansteenkiste, Sierens, Soenens, Luyckx, & Lens, 2009; Weinstein, Przybylski, & Ryan, 2012). In this respect, autonomy can be linked to the task of sense-making. Several studies emphasized the importance of autonomy in the freshman adjustment process, highlighting links with engagement, intention to persist, performance and actual retention (Hayenga & Corpus, 2010; Simons et al., 2004; Vallerand, Fortier, & Guay, 1997).

The need for relatedness encompasses the perception of closeness and friendships with one’s student peers (Vansteenkiste et al., 2009). It draws on the basic need to be connected, accepted and valued by others. In this respect, it can be directly related to the task of forging links with others. Several studies revealed the importance of social relatedness during the first year at university in student commitment, intention to persist and retention (Braxton, 2000; Hausmann, Ye, Schofield, & Woods, 2009; Robbins et al., 2004).

#### Adjustment Stage

Beyond the encounter phase, Nicholson depicted the *adjustment* stage, which encompasses concrete adaptation to the new environment (Nicholson, 1990). The core task of this stage is to reach a *“consonant relationship between the self and the environment”* (Nicholson, 1990, p. 88) by “melding” our behaviors to fit the requirements of the context (Nicholson & West, 1989). By contrast, the central danger is that of experiencing a person-environment mismatch accompanied by “degrading” and “grieving” experiences (Nicholson, 1990). According to Purnell (2002), the adjustment stage is expected to last for the rest of the first academic year.

This stage can be related to work on the student-institution fit (Bowman & Denson, 2014) inspired by the work of Tinto (2006). Such literature supports the need for the student to finely tune his or her actions and strategies in order to fit in with the demands of the university. In other words, the student’s behavioral and cognitive commitment to the educational context would help him to fit the context and to adjust.

According to Fredricks and colleagues (2004, p. 60), “cognitive commitment draws on the idea of investment; it incorporates thoughtfulness and willingness to exert the effort necessary to comprehend complex ideas and master difficult skills”. In the higher education context, this construct draws on deep-processing strategies: “thinking activities leading the student to focus on the underlying meaning and complex understanding of a task, such as relating, concretizing and critical processing” (Kember & Gow, 1994, p. 59) and self-regulation: “the ways in which an individual controls and directs his or her own actions” (Elias & MacDonald, 2007, p. 2518). Prior work in cognitive psychology has accumulated consistent empirical evidence for the finding that both deep learning strategies and self-regulation are crucial to understanding student learning and academic performance (De Clercq, Galand, & Frenay, 2013; Nota, Soresi & Zimmerman, 2004). For instance, Minnaert and Janssen (1999) have shown that self-regulation explains the same amount of variance in academic performance as do intelligence test scores.

Behavioral commitment relates to concrete participation in academic activities (Reschly & Christenson, 2012) such as attendance and study time. A vast body of literature substantiated the direct link between study time, attendance and academic marks (Robbins et al., 2004; Vandamme, Superby, & Meskens, 2005; Van den Berg & Hofman, 2005).

#### Stabilization Stage

The last stage of Nicholson’s model is *stabilization*, in which individuals are supposed to acquire *“sustained trust, commitment and effectiveness with tasks and people… to realize their potential in their roles”* (Nicholson, 1990, p. 89). Such a stage can be conceived as the equilibrium reached by the student when he fully adjusts to the academic context. As mentioned by Purnell (2002), this stage is barely reached during the first year at university. However, the student’s academic success could be a good sign of reaching the stabilization stage.

### Aim of the Study

Through qualitative real-time and post-hoc interviews, this study aimed at partially overcoming the lack of consideration of the multifaceted and temporal nature of academic adjustment. It also provided a qualitative investigation of Nicholson’s Transition Cycles model in the higher education context. More precisely, two main objectives underlie this approach: (1) to identify the key determinants and events of adjustment regarding the student’s experience; (2) to understand how these determinants and events interact in the adjustment process across the first year. To do so, this study poses the following two overall research questions:

1. *Which constructs are at play in the freshman adjustment process?*

The empirical literature and Nicholson’s model depict a cluster of constructs that are important to consider in the adjustment process. However, do these constructs really lie at the heart of the freshman experience? Moreover, little information is provided about the way these constructs act together on the adjustment process. In order to endorse a multifactorial approach, two more questions have been asked: How are these constructs intertwined? What is their precise role in the adjustment dynamic? Considering empirical evidence highlighting past performance, self-efficacy, behavioral engagement and self-regulation as major predictors of academic achievement, these constructs are expected to be important components of the adjustment process.

2. *How does the adjustment process dynamically unfold during the academic year?*

Temporal perspective is often left out of the picture in the empirical literature about higher education. Nicholson provides some hints on how to address this issue but many questions remain unanswered: What are the pivotal moments of the first year at university according to student accounts? When do the constructs take effect in the adjustment dynamic? Is there any recursive process to consider? According to Nicholson’s model and the work of Torenbeek and colleagues (2010) we would expect the beginning of the year to be a crucial moment of adjustment.

Such examination could establish guidelines for establishing a more global and dynamic framework of the freshman adjustment process at university.

## Method

### Participants

Semi-structured interviews were conducted in two stages over a period of two years with 17 freshmen (11 women; 6 men) majoring in Biology from the Faculty of Science of a Belgian University. This major was composed of 75 first year students. The participants were selected using one main criterion: the students should have made a direct transition between high school and university. In the Belgian educational context, the transition from high school to university is not always direct. Some teenagers decide to go abroad for one year between secondary education and university. Others change their major for biology. Others had already spent one year in the Biology major but had to repeat the year because they failed exams. Students with these profiles were not included in the selection process. Of the 75 first year students, 50 satisfied the criterion. Twenty-five participants were recruited randomly among these 50 students, by sending them an email which invited them to share their experience concerning the first year at university. Eight students were unresponsive or declined the request. The 17 remaining students were considered a sufficient number as the interviews provided significant saturation of the data.

Of the interviewed students, 2 passed the year, 6 failed the year, 4 dropped out of the program at the end of the year and 5 dropped out before the end of the academic year.^1^ The major in Biology is a particularly challenging context in terms of adjustment with an 81% average failure rate and 25% of freshmen leaving the program before the end of the academic year. In the Belgian educational system, the average failure rate is about 60% in the first year at university (ETNIC, 2015). This major was chosen in order to provide a context which vividly brings into focus student adjustment difficulties.

### Data Collection

Interviews were carried out in a quiet office of the Science department. Interview protocols were designed referring to guidelines provided by Willig (2013). Participants were first provided with an introduction to the study and ethical considerations. Then, several broad open-ended questions (e.g. “How do you concretely plan to tackle the rest of the year?”; “What were the major difficulties of your first year at university?”) were used as stepping stones to co-construct the interview with the participants (De Mol & Buysse, 2008). A thematic guide^2^ with several themes was also used to navigate in the students’ accounts and to steer the interviews.

In line with to the methodological recommendation of Zittoun (2009) a combination of post-hoc and real-time data was used. In the first stage (at the beginning of the first year at university/real-time), students were asked to describe their perceptions of the academic context; the difficulties experienced and the way they intended to overcome them. The average duration of interviews in stage 1 was 20 minutes. In the second stage (one year after stage 1/post-hoc), the same students were asked to give a retrospective overview of their first year; to describe the major events which fostered/hindered their adjustment to university and the way they behaved in order to succeed at university. These two complementary data collections provided a view of the process taking into account both spontaneous student explanation and reconstructions of their experience (Zittoun, 2009). In addition, based on the Leclerc-Olive figure (2002), students were asked to draw up a chart of how the first year unfolded.^3^ This representation was used as a support to discuss with the student the major events of the year. These figures are used to illustrate major events in the results section of this paper. The average duration of interviews in stage 2 was 70 minutes. The interviews were recorded and transcribed verbatim according to the guidelines of McLellan, MacQueen & Neidig (2003).

### Data Analysis

Data analysis was embedded in a critical realist epistemological stance in relation to the students’ accounts. According to Willig (2013), the aim is to get as close as possible to the research participant’s experience and to interpret it in order to further our understanding of the investigated phenomena. More precisely, the material was analyzed through two complementary methods: thematic and sequential analysis (Willig 2013).

*Thematic analysis* is a widely used method for identifying, analyzing and reporting patterns within data (Braun & Clarke, 2006). It was used to answer the first research question: “Which constructs are at play in the freshman adjustment process?”. In this analysis, the primary objective was to identify codes that capture the qualitative richness of the student’s accounts. To this end, the transcripts were read and emerging codes were assigned to segments independently by the first and the third author. This identification included a balance of deductive coding (derived from the theoretical framework) and inductive coding (emerging from a student’s account). A first analysis of the material was made by identifying “nvivo” coding as closely as possible from the student’s account. Then, in a second analysis of the material, nvivo coding was adjusted into theoretical coding allowing for our theoretical anchoring. Each code was labeled, defined and segmented in a coding manual in order to provide an effective tool of organizing segments. Memo-writing was used to spot the extracts that were difficult to interpret. In order to test coding reliability, the two coding manuals were compared, double-checked and discussed between the first and the third author in order to reach a mutual agreement and to refine the definitions of the codes (Braun & Clarke 2006). Next, the process of paraphrasing or summarizing each piece of data into themes and sub-themes was performed by connecting the codes and identifying themes through a discussion of the material between the first and the third author. This step entailed another reading of the materials in order to keep as close as possible to students’ accounts. A theme was considered as central for a participant when it was mentioned several times in both stages of the study. Finally, the second author revised and legitimated the themes identified by authors one and three by checking for internal homogeneity and external heterogeneity (Gion, Diehl, & McDonald, 2011).

**Figure 2:**
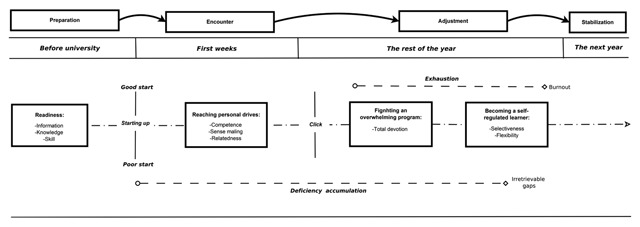
Temporal representation of the emergent themes and events from the analyses.

*Sequential analysis* interprets the course of the narrative in order to describe the sequential unfolding of the events. It was used to answer the second research question: “How does the adjustment process dynamically unfold during the academic year?” More precisely, it consists in the summary and the depiction of the major events experienced by the participants while taking into account their temporality. It complements thematic analysis by probing dynamic temporal perception of the interactions between the themes (Miles, Huberman, & Saldaña, 2013). To do this, a chronological summary of the experience of each participant was created by the first author. In order to adopt a helicopter view of the 17 experiences of the first year, the meaningful events of each participant, identified in the summaries, were inserted in a diagram^4^ inspired by the participants’ chart of the first year. These diagrams were analyzed in order to extract the major events related to the first year at university. In order to increase the validity of the analysis and our understanding of the material, investigator triangulation was also performed (Gion et al., 2011). Authors one and three re-analyzed the summaries together and discussed the potential difference of interpretation in the diagrams. As a final step in the interpretation a temporal representation of the results was crafted (see Figure 2).

## Results

Four major themes stood out from the thematic analysis and were particularly relevant for the participants as important constructs embedded in their adaptation process during the first year at university: readiness, reaching personal goals, struggling with an overwhelming program and becoming an autonomous learner. Each of these is composed of several sub-themes that determine their nature. Sequential analysis in turn revealed four significant and closely interrelated events in the first year that reveal the complex and dynamic nature of the adjustment process: a poor start; the insight/click; deficiency accumulation; and exhaustion. Finally, the overall analysis of the material was synthesized in a representation of student experience (Figure 2) which provides an example of the global dynamic overview of the themes and events identified. Figure 2 should not be understood as a strong conceptual model of adjustment but rather as a visual representation of the information gathered from thematic and sequential analyses. The descriptions of the results have been structured according to the temporal unfolding of this figure.

### Theme 1: Readiness

Readiness was identified as a first major theme. This theme was mentioned by 72% of participants and was central for 45% of them. Participants reported that the way they prepared to face the first year at university was crucial to their adjustment to university in three ways: information, knowledge and skills. These three sub-themes are close to the tasks of preparation as described by Nicholson (1990).

*Information*. First, the information process undertaken in order to choose the study program allowed the student to finely tune his/her expectations about the characteristics of the new academic context in terms of difficulty, requirements, workload and so on. I041: *“Concerning the workload, it is roughly as I imagined because I had been warned by everybody: ‘yes, you are crazy to register in this program, it is extremely hard”*. Other students reported the danger of a poor study choice process. They shared their initial misconceptions and the important gap between their expectations and the actual program. I012: *“It is really different from what I expected. I thought that when you are at university, you party a lot, you only attend few courses and everything is going alright but it is very far from the actual reality”*.

I072: *“I had another vision of university and of the Biology major, it was not how I expected it, I didn’t feel in the right place”*. These testimonies substantiate the importance of developing precise expectations about university as assumed by Nicholson’s model (1990). Moreover, they also support empirical work on the study choice process (Biémar et al., 2003; Germeijs et al., 2012; De Clercq et al., 2016).

*Knowledge*. Second, the mastery of core required *knowledge* of content and theories inherent in the program is also identified as important in participants’ interviews. Previous relevant secondary education eases the early adjustment to the new educational context and prevents students from needing to fill in initial theoretical gaps. I022: *“I tried to study what I can but the understanding of several courses was totally dependent on mastery of previous content. So, I was kind of stuck, particularly in Mathematics where I didn’t understand anything. Mathematics was not my major in high school so…”*. This is consistant with previous studies on the impact of past performance on students’ adjustment to higher education (DaDeppo, 2009).

*Skills*. Finally, the mastery of study *skills* related to autonomous learning also emerged from the students’ interviews. Study skills acquired during secondary education give students the tools to effectively meet the requirements of the new learning environment. If autonomous learning skills have not been acquired before entrance to university, the student has to develop them during the first year while coping with other program requirements, which could hinder the chance of success. I132: *“What makes it go wrong is my lack of preparation. I ended high school with insufficient background. I had no working method. They taught us no working method, no note-taking, no study strategy, we didn’t know. In high school we just have some sheets, little chapters, it was easy”*.

### Event 1: Starting Out

**Figure 3:**
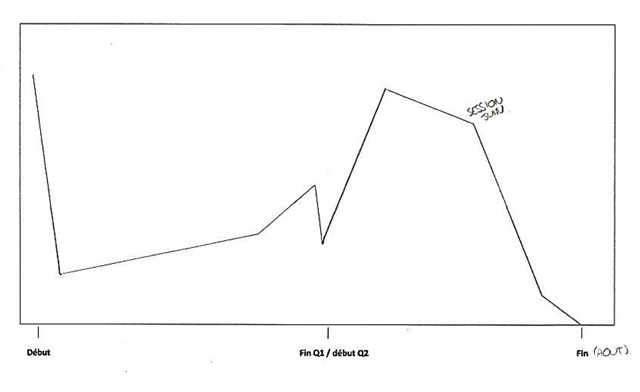
Participant 17 schematization.

Directly arising from student readiness, a first event was identified in the sequential analysis: *starting out*. The participants particularly emphasize the importance of the first weeks at university. A positive attitude at the beginning of the year seems essential for students to thrive in the academic context. In order to make a good start, student readiness plays an important role in early attitude and mindset. Information choice process prevents students from being shocked by the new learning context and helps them to quickly cope with the requirements of the program and to avoid the inherently poor start of an inadequate choice process. Moreover, students’ initial knowledge and skills are essential to facing new courses without initial deficiencies. Participant 17 illustrated the important drop in his or her motivation due to a poor start, shown below (Figure 3). I172: *“At the very beginning of the year I was really motivated and I thought that I could pass the year. But, after 2 weeks, I was already lost; I didn’t understand anything in any courses anymore.”* This stage can be linked to the encounter stage of the transition cycle (Nicholson, 1990) and exemplifies the importance of preparation in managing the shock of the new context.

### Theme 2: Reaching personal goals

Participants reported the importance to them in terms of confidence, passion and belonging of performing in the academic context. This second major theme was mentioned by 81% of participants and was a central theme for 36% of them. This theme closely resembles the tasks from Nicholson’s encounter stage (Nicholson & West, 1989) and is embedded in self-determination theory (Deci & Ryan, 2000).

*Gaining Confidence*. Participants emphasized *confidence* as a central construct in the adaptation process. Lack of confidence and feeling powerless can impede student involvement.

I112: *“I know what I have to do to succeed but, I don’t feel able to do it. I have no self-confidence… I am so afraid to fail that it blocks me. It is the fear of failing while you are really trying to succeed.”* However, unbalanced feelings of confidence from one course to another can also be detrimental. Students often privileged commitment to courses where they felt confident in their abilities to succeed at the expense of other courses.

*Making sense*. Students also reported the importance of courses *making sense* to them and of being passionate about them as a central means of sustaining the effort and involvement necessary for success. I042: *“It is important for our future job to learn theories, formulas and to know how to use a microscope… We will always have a need for what we are studying now…and the courses are very interesting. I’m glad to learn what I learn… I love my program more and more every day.”* I012: *“I love to study cells; I am really passionate about it. Courses are very interesting. So, I study for pleasure and I’m glad to learn what I learn…, I love my program more and more every day”*. Just as for competence, and despite the importance of the value of the courses, an unbalanced perception of the value of different courses can be detrimental to the student’s adjustment. Some courses are overvalued and overinvested to the detriment of other, often more difficult, courses. I082: *“Given that I didn’t like the course, I put it aside and that was my biggest mistake.”*

*Forging Links*. Finally, the *sense of belonging* was extracted from the data. Students reported the need to socialize and forge links with peers and the department. However, belonging to an adaptive social group that fosters involvement in academic tasks is necessary. In this new social context, students emphasized the influence of other students in their conceptions of, and actions and reactions to, the academic context. I032: *“I really think that I’m finally starting to study and attend the courses thanks to my friends. They gave me a hard push, it was really motivating”*. Students also emphasized the potential negative effect of their belonging to a social group holding them back. I112: *“We were all in the same situation. It was a mass effect. Somebody said ‘let’s have a drink’ and everyone followed. We dragged each other down and we all failed the year.”*

### Event 2: The Click

**Figure 4:**
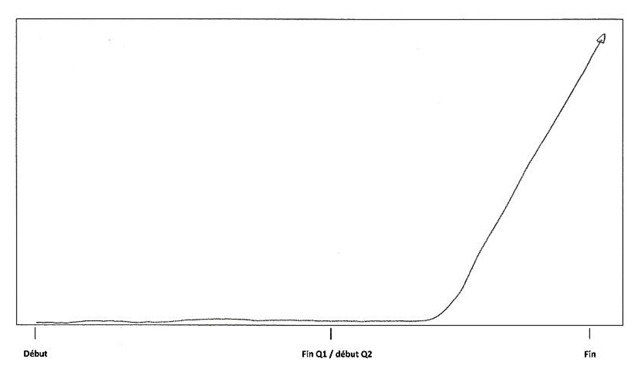
Participant 3 schematization.

According to the Nicholson transition cycle, making sense of the context, feeling competent to cope and forging links with others are the tasks necessary to moving smoothly from the encounter to the adjustment phase (Nicholson, 1990). However, participants also described another important event beyond the three above-mentioned drives. This was identified as the second event of sequential analysis and was in vivo coded^5^ as *the click*. Several students demonstrated a rigid and passive attitude concerning the critical requirements of the academic context. In contrast to persistent students, they passively endured the difficulties of the year without trying to find a more effective way to get through it. *I022 “It is not easy to develop the necessary energy, it takes time, it is very hard. So I felt overwhelmed by the events, I passively endured and that’s it.”* What really mattered for the students was the sudden realization that they had to change their attitude in order to give their utmost to face first year difficulties and to achieve. This shift in mindset seems very difficult to adopt for freshmen. They reported the necessity of achieving an insight, of triggering a click so as to change their state of mind. This raised awareness is often activated by actual, formal and “objective” feedback such as, for example, the results of a test. Such an event is exemplified by participant 3’s material and visual representation, which testify to his or her huge increase in involvement (Figure 4). I032: *“When I received my disappointing exam results, I started to work. Yes, I really started to work. I realized that if I wanted to pass, it was time to move forward. It was just like a huge click inside of me.”*

### Theme 3: Fighting against an overwhelming system

Engagement in the educational context was found to be a central source of difficulties for the participants. Every student mentioned this third major theme and it was identified as central by 72% of them. The program is characterized by a *heavy workload* and *fast work pace*. Moreover, understanding of the courses’ content is contingent on mastery of previous knowledge. Students who struggle with understanding a course can quickly become overwhelmed. I072: *“It’s completely demotivating. As soon as we miss something, it becomes very difficult to catch up later. So, it accumulates and accumulates, and we finally realize that it will be impossible to pass the exam. It’s irretrievable.”* If the student is left too far behind, it will be almost possible to make up the gap. Students thus constantly need to devote a *large amount of time and energy* to study and make it their top priority in order to keep up with the pace. I041: *“I work every day and every weekend. When I go home, I work 2 hours until dinner. During the weekend, I work all Saturday long and on Sunday morning.”* The first year is depicted as a constant fight to “keep your head above water”. This theme can be related to Nicholson’s task of the adjustment phase as the student’s necessity to mold their behavior to fit the program requirements. It also echoes the central role of behavioral engagement in the adjustment process (Sandler, 2000; Soria & Stebleton, 2012).

### Theme 4: Becoming a self-regulated learner

Another theme was identified in 63% of participants (central for 36%): the necessity of *becoming a self-regulated learner*. Lots of students reported working a lot without any relevant feedback on their performance. The *flexibility and selectivity* of learning strategies and engagement stand out from the data as being central to adjusting to the requirements of the heavy workload. Students pointed out the necessity of adopting a highly effective strategic management of their study program. As illustrated below, some students reported a high selectivity of their engagement based on and self-awareness and on a course’s demands. I032: *“Concerning the integration project, I think I had one of the lowest marks of the class. Even if it was very interesting, it demands an enormous investment for a very small course. It isn’t worth it”*. I112: *“I am usually too self-demanding and it is difficult to tell to myself ‘it is easy, you have to stop studying’. But when I run out of energy, I can still tell myself ‘Stop, have a break, you’ve mastered it very well now’”*. Conversely, several students reported difficulties in leaving behind their usual study strategies and adopting this flexible and selective way of learning. I082: *“I didn’t finish all my courses’ reports in time; I don’t even know how I could have done it. Moreover, studying only my reports was not enough, it was unrealistic…”*. Such results confirmed the importance of considering both the quantity and the quality of engagement. More precisely, this finding supports the role of self-regulation in the conceptualization of the process of academic adjustment to the first year at university (Zimmerman 2005). It is also worth noting that the key factor is not the type of strategy used but rather the fit between the strategy and the course expectations.

### Event 3 & 4: The balance between deficiency accumulation and exhaustion

**Figure 5:**
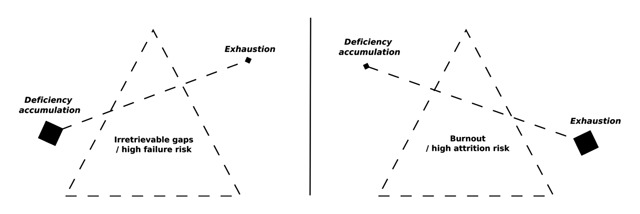
The delicate balance.

Two interconnected events were also identified in sequential analysis which disclosed a recursive cycle linked to the two major themes: fighting against an overwhelming program and becoming a self-regulated learner. This relation is illustrated in Figure 5, below.

**Figure 6:**
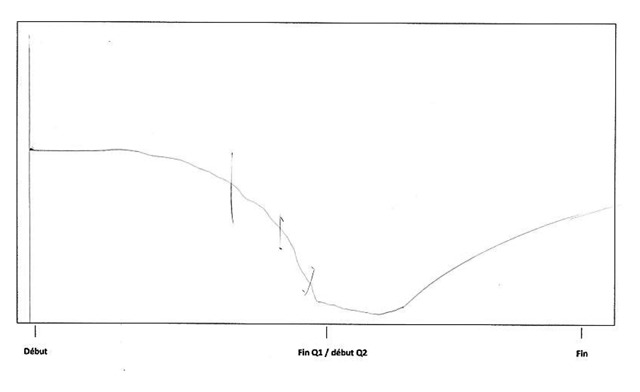
Participant 7 schematization.

**Figure 7:**
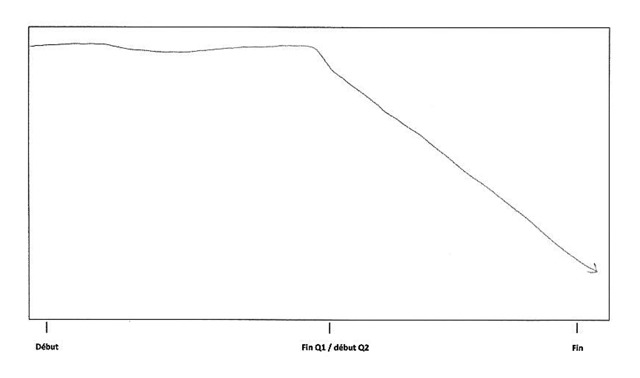
Participant 8 schematization.

The first event is students’ *exhaustion*. This progressive state could result from an imbalance between involvement and remaining resources. The demanding academic context requires an intensive engagement which can quickly turn into student exhaustion if it is not managed by self-monitoring and effective autonomous learning. Students with poor self-regulation abilities desperately try to keep up with the pace and progressively run down their energy resources until they reach a state of exhaustion. Participant 7 highlighted this progressive drop in energy leading to exhaustion in Figure 6, below. I072: *“The first three days of the exam session, I slept because I couldn’t stand it anymore… I should study more but I was physiologically and psychologically unable to do it.”* On the other side of this constant fight to keep their head above water is *deficiency accumulation*. If a student is not completely dedicated to his/her program or if he/she is not efficient enough, deficiencies will start to accumulate. The more students endure this situation the less they will be able to reverse the trend because existing deficiencies impede the understanding of new concepts and theories, which leads to the occurrence of new gaps in the courses. Some participants describe it as a “snowball effect”. After several months, these students are left too far behind to overcome their gaps in mastery of the core content and competencies of the avoided courses and progressively drop out from university. Such a final decision is often taken after the first exam session, as represented and described by participant eight (Figure 7). I082: “*In January, I passed no exams… frankly, it’s sad, I didn’t expect to fail the session so brutally… At the beginning of the second semester, I didn’t know how to restart… I was totally overwhelmed, it was impossible to fill the gap.”*

## Discussion

Transition to higher education is a topic of substantive investigation but the global understanding of this temporal process remains relatively undocumented. This study provides a stride in this perspective by providing a multifaceted and dynamic understanding of first year experience and by vividly exemplifying this experience through students’ discourse. Despite the inherent limitations on generalization from qualitative research, our work provides a means of thinking about the specific transition context of the first year at university of a Belgian biology major. This study could offer relevant elements for reflection concerning the literature about first year experience.

### A process modeling of academic transition

The importance of several highly documented variables in the freshman adjustment process was highlighted in this study. The meta-analysis of Richardson and colleagues (2012) demonstrated the importance of past performance, self-efficacy beliefs, study time, self-regulation, motivation and strategic approach to learning. A large proportion of the student’s accounts can be related to these factors. Moreover, the relevance of self-determination theory (Deci & Ryan, 2000) and the Nicholson Transition Cycle (Nicholson, 1990) has been corroborated. As shown in the results section, the essential tasks described by the model of transition cycles can be linked to the major themes extracted from the analysis. For example, the students emphasize the importance of readiness, of making sense or of feeling competent. Yet the major interest of the results lies in the multifaceted perspective disclosing the way these constructs are involved together in the adjustment process. In this respect, several conclusions can be drawn which question traditional empirical results from single-factor approaches and the way transition is depicted by Nicholson’s model.

### Bringing important nuances to higher education literature

First, complex relations between the constructs appeared in our specific context of investigation. A striking example is the relation between behavioral and cognitive engagement. The students added important nuances to the role of involvement by revealing that a certain quantity of work is necessary but is not enough to succeed if it is not combined with good quality of work. Without flexible management of their engagement students were doomed to exhaustion. Such a finding is in line with the postulate of Reschly and Christenson (2012) which asserts that the impact of behavioral engagement on academic achievement will depend on the student’s cognitive engagement. These results particularly highlighted the limitations of isolated investigation of these constructs and the need for a more integrated perspective previously pointed out by Allen and colleagues (2010). A further clarification arose regarding cognitive engagement. According to the Biology freshmen, the key to success was not the type of strategy used but rather the management of the fit between strategy and course expectations. An in-depth understanding of the subject can be an ineffective strategy for achievement when the course required only superficial learning. This result echoes the work of a number of authors (De Clercq et al., 2013; Michaut, 2012; Vermunt, 2005) which insists on the importance of considering the specific features of course requirements in the investigation of the effect of learning strategies on achievement. It also confirmed the importance of effective self-regulation strategies for the participants. Beyond learning strategies, the ways in which the students monitored, regulated and controlled their learning appears to be crucial in managing their highly demanding academic context. Such a finding is in line with Nicol’s claim (2009) that students’ self-monitoring and evaluative judgments about their own work are of the utmost importance in effectively coming to terms with the demands of the academic environment. As a global framework, the transition cycles model did not tackle these complex relations. The tasks are expressed in very general way (e.g. melding our behaviors to fit the requirements of the context) which eludes these specificities.

Second, it is worth noting that some of these constructs had ambivalent effects on the student process. For instance, too much passion or confidence in one course can entail underinvestment in other less valued, but still important, courses and endanger the student’s overall academic success. These results do not fit with the traditional investigation of motivational variables; such studies consist in the overall analysis of the impact of motivation in a program without considering its variations from one course to another (e.g. Fenollar, Romajn, & Cuestas, 2007; Vallerand et al., 1997). Such an approach could conceal the potentially deleterious effect of unbalanced self-confidence and perceived course value within the program and lead to the conclusion that an increase in course value and self-confidence always has a beneficial impact on adjustment. It is worth noting that this issue is also applicable to this study. Even if the participants spontaneously tackled the importance of the variation of motivation between the courses, our investigation does not systematically take into account course specificities. Other ambivalent effects could therefore have remained hidden. Such an issue needs further consideration in the literature.

Third, the student testimonies emphasized the necessity of considering the qualitative side of the constructs beyond their quantitative aspect. For example, students’ accounts revealed that high social integration is not good per se; integration in an ill-adapted social group could in fact impede a student’s adjustment. This effect may be stronger in the first-year transition period when students would be particularly influenced by the attitudes of peers (Dennis, Phinney, & Chuateco, 2005). Our findings support the insight that the quality of integration may be more important than the quantity of it, as suggested by Rubin’s meta-analysis (2012). Such nuances raise new questions in our understanding of the role of student integration (Tinto, 2006) and emphasize the limitations of the current quantitative measures of this concept. In this respect, the framing and validation of enriched integration scales considering the quality of social groups could be an important step forward. The potential ambivalent effects highlighted above are also overlooked by the transition cycles model, which does not really address this question.

### A temporal perspective on academic adjustment

A second contribution of the present study lies in the temporal conceptions of the first year at the university, mainly neglected in the literature to date. For example, Biology students highlighted the snowball effect of deficiency accumulation resulting from ineffective management of academic demands. When actively facing this recursive problem, students were also confronted with progressive exhaustion, which may lead to burnout if it is not effectively managed. Such results show the relevance of a more dynamic perspective of academic adjustment.

In this respect, Nicholson’s model of transition appears to provide an interesting framework for understanding the temporal nature of adjustment. Several events highlighted by the students closely resemble Nicholson’s stages of preparation, encounter and adjustment. However, a major limitation of this model can be identified in the total disjunction between the encounter and adjustment phases. Nicholson conceptualized these stages as clearly disjunctive (Nicholson & West, 1989). The tasks and pitfalls of a stage are supposed to be confronted in order to move to the next stage. The students’ accounts in fact support the temporal sequence of Nicholson’s stages. However, they also revealed that the tasks and pitfalls of one stage did not stop at the beginning of the next. For example, students emphasized that the need to feel competent, make sense and relate to others is a continual challenge throughout the year. The adjustment stage could therefore be conceptualized as the stage where the student molds his/her behavior to fit the environment while maintaining a high feeling of competence, autonomy and relatedness.

### The characteristics of the context: a necessary consideration

Another conclusion that can be drawn from this study is the important role of the context in the adjustment process. In terms of our results, the main challenge for the freshman is to cope with the specific demands of his/her study program. These results concur with the theoretical importance of the context in the adjustment process (Tinto, 2006; Lizzio, Wilson, & Hadaway, 2007). Such consideration is often empirically overlooked in higher education literature. Some studies tried to consider this question through multi-level analysis (Van Der Hulst & Jansen, 2002; Van den Berg & Hofman, 2005) and showed that around five percent of variation in achievement is due to the curriculum. However, more research is needed to provide a clearer picture of the importance of the context in adjustment issues (Kuh, Kinzie, Schuh, & Whitt, 2011). Neither is context specificity addressed by Nicholson’s model, which provides a universal framework for transition.

Such a consideration implies that the results of this study are, to some extent, peculiar to the specific features of the investigated context (in this case, a major in Biology from the science faculty of a Belgian university). This consideration emphasized the necessity to consider these results carefully and to restrict the transferability of the conclusions to a different context. Hence, remarks, comments and assumptions formulated in this conclusion have been intended to encourage further reflection on the first year at university but not as generalizable truths.

This finding also raised questions regarding the universal nature of the academic adjustment process. Some authors have highlighted the fact that there is substantial variation in the determinants of achievement from one study program to another (De Clercq et al., 2013). In this regard, new questions can be asked: “To what extent is the adjustment process determined by the specificity of the study program?”, “What are the universal vs context-related determinants of adjustment?”, “To what extent can literature about the first year at university be conceived of as a coherent whole?”. Such questions deserve more attention in further studies in order to clarify the global nature of academic adjustment.

### Questioning the Model of Transition Cycles

With regards to the results of the findings, the legitimacy of using a global theoretical framework such as the Nicholson Transition Cycle model (1990) can be questionned. As expressed by Purnell (2002), the transition cycle model has great potential to help us develop an overall dynamic picture of adjustment. This assumption was corroborated by our results, which showed that Nicholson’s assertions and stages were in line with students’ concerns and testimonies. However, this model demonstrated several limitations concerning the in-depth understanding of the transition process. It does not take into account the context specificity, the complex interrelations and the ambivalent effects of the construct composing academic transition. Does this mean that the transition cycles model is not an adequate tool for tackling the adjustment issue? We believe that these limitations do not impair the validity of this model as a global conceptual framework. The same limitations can be pointed out in a number of global frameworks, such as self-determination theory (Deci & Ryan, 2000) or Tinto’s attrition model (2006). Nicholson’s model can be viewed as an adequate but incomplete frame of understanding. This study reminds us that the fit of a global framework to a specific context is instrinsicaly limited and needs to be supplemented with a careful consideration of the micro-processes (complex interrelationships, ambivalent effects, …) occuring in a specific situation.

### Limitations of the Study

Several limitations of the study have already been stated, such as the specific details of the context investigated or the lack of consideration of courses features. However, two additional limitations should be noted. First, the method used to address the dynamic nature of adjustment can be questioned. As suggested by Zittoun (2009), this study used a combination of real-time and post-hoc data in order to capture student change from a prospective and retrospective point of view. However, this perspective could have been complemented by a longitudinal design following students over several consecutive months in order to capture the developing patterns in their behaviors, perceptions and attitudes. As suggested by Willig (2013), the use of diaries could offer such a longitudinal complement and better capture the time dimension of transition. Second, the selection procedure could also be questioned. Students were selected on a voluntary base and some of them declined our invitation to participate in the inquiry. Such a procedure implies selection bias. Participants were students motivated to share their experience and to reflect upon their own adjustment process. In this respect, it is possible that certain student profiles couldn’t be reached by the present study. One way of overcoming this limitation would have been to select students based on their entrance characteristics (motivation, prior knowledge, self-regulation competence, achievement level, …). However, such a mandatory procedure could have led to other limitations such as damaging the quality of the testimonies (Willig, 2013).

### Some suggestions for action

Embedded in the analysis of the material, several moments and elements have been pointed out to promote freshman achievement. Both our results and Nicholson’s model (1990) suggest that significant energy should be devoted to the preparation stage before university entrance. In this stage, high school training plays an important role, as shown by studies on past performance (DeBerard et al., 2004). However, there is still work to be done on students’ entrance study skills. The metacognitive literature suggests that there is also important work yet to be done on the promotion of self-regulated behavior before and during the first year (Zimmerman, 2005). Such work would maximize the effectiveness of students’ work and prevent some of them from suffering a poor start, deficiency accumulation and exhaustion. Following Nicol’s results (2009), an effective way of promoting self-regulatory strategies could lie in formative assessment and feedback practices. For example, “the integration of opportunities for reflection and self and peer assessment in learning are beneficial as they provide students with early experiences of self-monitoring” (Nicol, 2009, p. 336).

Another important intervention also lies in vocational guidance. Guidance is often focused on a long-term career plan, which has been shown by the literature to be an important source of motivation (Germeijs et al., 2012). However, our results particularly highlight the necessity of providing students with concrete and clear information about the academic context. To do so, high school students could be encouraged to experience university before choosing their major. Such regular experiences would lead to clear and realistic expectations, thus easing the shock of the encounter with the new academic context.

A second period, one that is often neglected, is the encounter stage or the first weeks at university. Several authors have emphasized the importance of the first week of transition from secondary to higher education (Neuville, Frenay, Noël, & Wertz, 2013; Sauvé et al., 2007). More precisely, Purnell (2002) particularly insists on the huge potential of this stage to act on students’ perceptions and beliefs with lasting consequences for his or her confidence, sense-making and relatedness. This postulate is supported by studies in social psychology (Yeager & Walton, 2011) which have pointed out that a brief intervention in a sensitive phase such as the first week at university can have important long-term effects on student adjustment. For instance, Walton and Cohen (2011) highlighted the lasting effects of brief social-belonging intervention on well-being and academic achievement over a three-year period for some minority students. Such a perspective therefore deserves to receive more attention in educational literature.

Finally, the results disclosed snowball effects which constitute a plea for the early fostering of academic adjustment. If insight is triggered too late in the year, students have often accumulated too many deficiencies to reverse the current trend. A concrete way to act on this dynamic could be by providing students with early feedback on the way they are coping with the new context (Núñez et al., 2015). Such feedback could trigger a change in the mindset and attitude and foster their engagement in the context. One facet of the literature supports the importance and richness of informal and formal feedback in the educational context (Young, 2005).

To conclude, this study supports the view that ‘the whole is greater than the sum of the parts’ (Willig, 2013). Indeed, results drawn from the present analysis showed that the adjustment process is complex and that its constituting parts show dynamic relationships that are interdependent. Moreover, a number of dynamic recursive circles have been identified that lead to disengagement and the accumulation of irretrievable gaps, as such destroying any chance of passing the year. Therefore, the student adjustment process cannot be seen as the sum of adaptive factors, but should rather be seen as a complex recipe where each ingredient needs to be taken into account and accurately measured.

## Additional Files

The Additional files for this article can be found as follows:

- pb-58-1-409-s1.pdf
  **Appendix 1** Interview protocols. DOI: https://doi.org/10.5334/pb.409.s1
- pb-58-1-409-s1.pdf
  **Appendix 2** Figures adapted from Leclerc-Olive (2002). DOI: https://doi.org/10.5334/pb.409.s1
- pb-58-1-409-s1.pdf
  **Appendix 3** Illustration of a participant scheme. DOI: https://doi.org/10.5334/pb.409.s1

## Footnotes

In the Belgian educational system, students pass the year if their average final grade is above 10 out of 20.

An illustration of the thematic guide is in the appendix.

An illustration of the Leclerc-Olive figure is in the appendix.

An illustration of these diagrams can be found in the appendix.

In vivo coding refers to a sequence directly coded with the words of the participants in order to avoid importing theory into the analysis (Willig, 2013).
