# Supplementary material for: The Delicate Balance to Adjustment: A Qualitative Approach of Student’s Transition to the First Year at University
Source: Psychol Belg. 2018 Apr 27;58(1):67–90. doi: 10.5334/pb.409 (PMC6194531; doi:10.5334/pb.409)

Appendix

Appendix 1

Interview protocol T1:

- You are in a major in Biology. Could you explain this study choice to me?
- How did you imagine this major before entering at the university? What is different now?
- How is your study going?
- Do you feel well adjusted to the university? Could you elaborate on it?
- Do you experience any difficulties since the beginning of the year?
- How do you tackle these difficulties?
- Which resources do you use to adapt to the academic context?
- How do you concretely plan to tackle the rest of the year?”
- Can you conciliate your studies with the other aspects of your life? How?
- Do you think that you are on the right track to pass the year? Why?
- How do you feel during the lessons?
- If you could start this academic year over, would you change anything?
- How important are your study to you?
- Do you want to add anything else?

Interview protocol T2:

- How did this first academic year go?
- How do you explain your performance?
- How do you feel right now when you think to your last year?
- What were the major difficulties of your first year at university?
- One year ago we discussed about your experience at the university. Do you think that something changes in your mind since then?
- Could you explain your chart to me? What are the major events of this first year?
- How did you feel during the lessons?
- Are you satisfied with your first year? Could you elaborate on this?
- How did you do to organize your studying?
- Do you feel well adjusted to the university? Could you elaborate on it?
- Which resources do you use to adapt to the academic context?
- Could you conciliate your studies with the other aspects of your life? How?
- How important are your study to you?
- Do you want to add anything else?

Appendix 2

Figures adapted from Leclerc-Olive (2002) and used during the interviews in order to make participants sketch their first year experience.

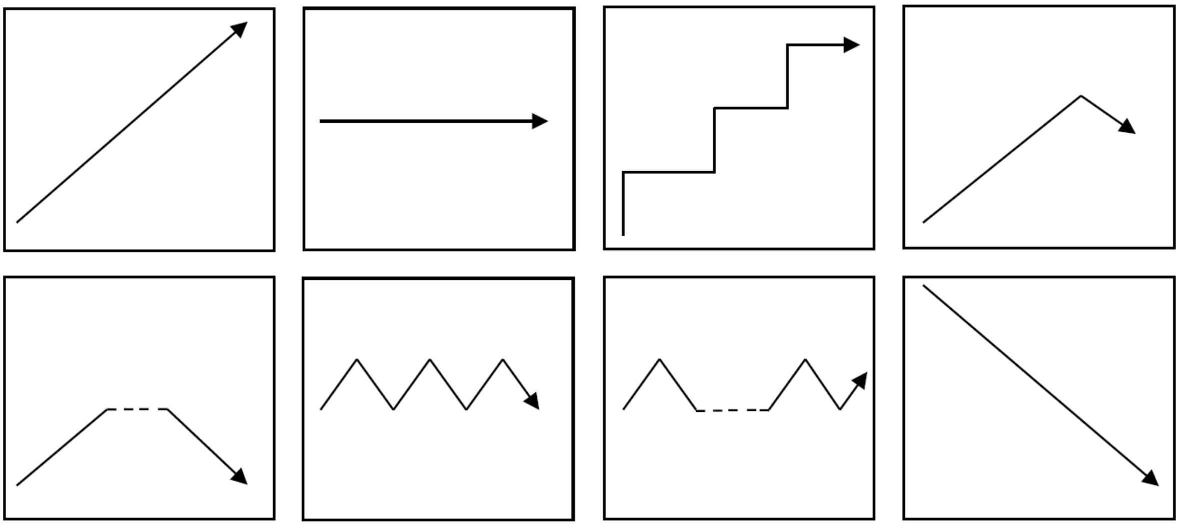

Appendix 3
Illustration of a participant scheme

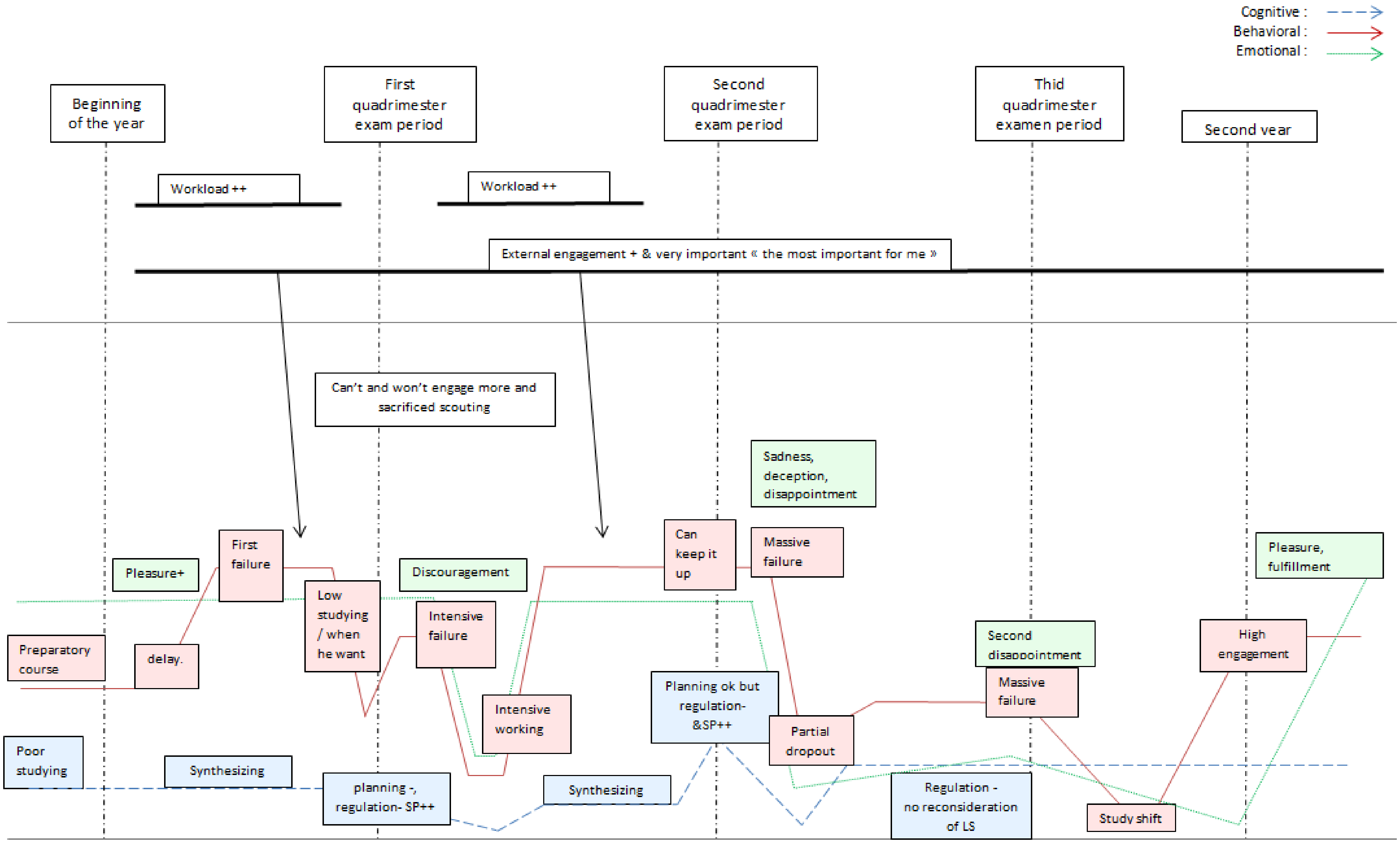

Supplement: Supplementary file 1 [file pb-58-1-409-s1.pdf]
